# Radiographic Changes to Silver Diamine Fluoride Treated Carious Lesions after a Rinsing Step

**DOI:** 10.3390/dj10080149

**Published:** 2022-08-09

**Authors:** Zaher Jabbour, Maryam Esmaeili, Marc Hayashi, Reuben Kim

**Affiliations:** Restorative Materials Research Laboratory, Section of Restorative Dentistry, School of Dentistry, University of California, Los Angeles, CA 90095, USA

**Keywords:** silver diamine fluoride, potassium iodide, water, rinse, caries

## Abstract

Silver diamine fluoride (SDF) is radiopaque. This in vitro study compares the changes in the radiopacity of carious lesions after SDF application, potassium iodide (PI) application, and water rinse. Ten recently extracted human teeth were sectioned and divided into two groups (*n* = 10 in each group): Group 1 = SDF, Group 2 = SDF + PI. Teeth in Group 1 received SDF for 1 min and rinsed with 15 mL water. Group 2 received the same protocol with the addition of PI application for 1 min after SDF application. All samples were scanned with micro-computed tomography before SDF application, after SDF application, after PI application (group 2) and after water rinse. The radiopacity of the carious lesions increased significantly after SDF application in Group 1 and 2 (*p* < 0.017, *p* < 0.008, respectively). A significant increase in radiopacity after PI application was also observed in Group 2 (*p* < 0.008). Water rinsing significantly decreased the radiopacity in Group 1 and 2 (*p* < 0.017, *p* < 0.008, respectively), but the radiopacity remained significantly higher than the preoperative values (Group 1 *p* < 0.017, Group 2 *p* < 0.008). The radiopacity of carious lesions increases after SDF and SDF + PI applications. Water rinsing could reduce the radiopacity of SDF and SDF + PI treated carious lesions, and might reduce the content of SDF in carious lesions.

## 1. Introduction

Treatment of dental caries has historically focused on the surgical removal of carious lesions and replacement of tooth structure with biocompatible materials such as dental amalgam, resin or glass ionomer. Silver Diamine Fluoride (SDF) has been used in several parts of the world as a non-surgical treatment to prevent and arrest dental caries [1]. In 2014, SDF was approved by the Food and Drug Administration (FDA) as an anti-sensitivity agent. Although the mechanism of action of SDF is not fully understood, the silver in SDF displays antibacterial properties, whereas the fluoride in SDF promotes the remineralization of hydroxyapatite in enamel and dentine [2,3].

Current guidelines by the American Dental Association encourage the use of non-surgical methods such as SDF as viable alternatives for the management of dental caries [4]. SDF seems to have potential beneficial effects in slowing the progression and arresting dental caries [4]. SDF offers many advantages including low cost and ease of application, especially in children [5]. Conversely, several disadvantages are associated with SDF application such as dark staining of carious lesions, likely due to the silver content, possible fluoride ingestion, and decrease in the bond strength of adhesively applied restorative materials [3,6].

Multiple protocols for SDF application were reported in the literature. Although the manufacturer recommends only blot drying the tooth after SDF application, others have recommended trying to keep the tooth isolated for at least 3 min [5,7]. Nonetheless, many clinical situations require application of restorative materials (e.g., composite resin and glass ionomer) following SDF to seal the carious lesion or to improve esthetics. Application of these materials necessitates a rinsing step to eliminate the phosphoric acid or the polyacrylic acid. In addition, water rinsing after SDF application was proposed to restore the bonding strength of restorative materials [6,8]. Furthermore, Potassium Iodide (PI) rinsing after SDF application was advocated to eliminate silver-mediated dark stains [9]. However, it is unclear if the rinsing with water or PI could reduce the SDF content within the carious lesions. Therefore, the aim of this study is to quantify the amount of SDF in carious lesions before and after rinsing with PI and water.

## 2. Materials and Methods

This in vitro study was approval by the Institutional Review Boards of the University of California Los Angeles (IRB #19-002271). A total of ten recently extracted permanent teeth with large carious lesions and no previous restoration were sectioned in the coronal plane into two halves (two groups, 10 samples in each group): Group 1 (SDF group) treated with SDF only, then rinsed with 15 mL water, and Group 2 (SDF + PI group) treated with SDF followed by PI, then rinsed with 15 mL water.

### 2.1. SDF and PI Application

All teeth were treated with 38% SDF (Advantage Arrest, Elevate Oral Care, West Palm Beach, FL, USA) according to the manufacturer recommended protocol. Briefly, teeth were cleaned of gross debris with a spoon excavator and dried with a cotton pellet. SDF was dispensed into a disposable dappen dish and applied directly onto the area of carious lesion using a microbrush applicator for 1 min. Samples in Group 2 received the same protocol as samples in Group 1 except that a saturated solution of potassium iodide (30 mg/drop) (Chimdirect, Stockbridge, GA, USA) was applied for 1 min after SDF application then rinsed with 15 mL water.

### 2.2. Micro-Computed Tomography (Micro-CT)

**Figure 1:**
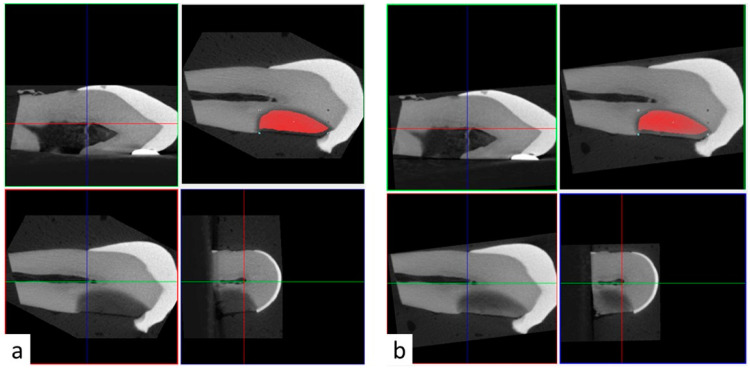
Three dimensional coregistration of reconstructed 3D images using DataViewer software. (**a**) Preoperative micro-CT images showing the region of interest (red outline) adapted to the carious lesion. (**b**) Coregistered 3D micro-CT images after application of a radiopaque material (e.g., potassium iodine) with the same region of interest transferred from preoperative to post-operative images.

All teeth samples were imaged in air settings using micro-CT (SkyScan 1172; Bruker, Kontich, Belgium). Images were acquired using 11-Mp digital detector. Samples were scanned with 70 KV, 142 microamperes, 12 micron resolution, 0.4 rotational step and 1 mm aluminum filter. Samples were scanned preoperatively before SDF application, after SDF application, and after rinsing with 15 mL of water. Samples in Group 2 were also scanned after PI application. Three-dimensional reconstructed images were superimposed using the 3D coregistration function in DataViwer software. Briefly, the pre-operative reconstructed images served as reference images and stayed stationary. Post-operative reconstructed images (target images) were repositioned and registered in three dimensions over the reference images (Figure 1). As a result, it was possible to transfer the same region of interest (ROI) from the pre-operative reference images to the post-operative target images (Figure 1). The ROI was selected based on the radiopacity of the carious lesion in the preoperative 3D reconstructions using the thresholding function based on 0–255 greyscale units. The upper and lower threshold limits were set to allow clear distinction between the radiopacity of the carious lesion and the surrounding intact dentin, and between the radiopacity of the carious lesion and the surrounding wet gauze, respectively. These parameters were relatively consistent among samples with the upper limit ranging between 95–115 greyscale unit, and the lower limit ranging between 40–55 greyscale unit.

### 2.3. Statistical Analysis

Descriptive analyses of data were presented as boxplots showing the median and interquartile range. Wilcoxon Signed Rank Test was used with Bonferroni adjustment for multiple within-sample comparisons. Mann–Whitney U test was used for between group-comparisons at baseline and after SDF application. IBM SPSS software (IBM Armonk, New York, NY, USA) was used for all statistical analyses with *p* < 0.05.

## 3. Results

**Figure 2:**
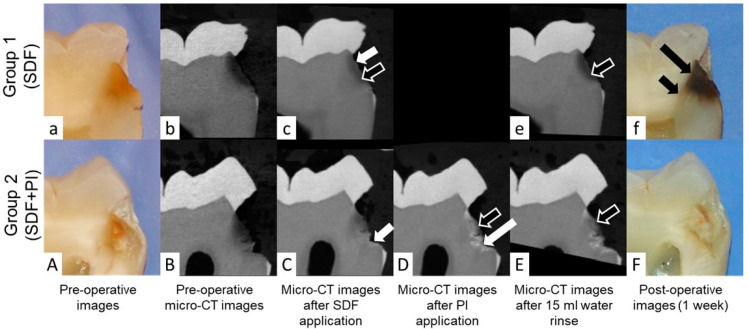
Pictures and coregistered 3D micro-CT reconstructions of sectioned teeth in Group 1 (SDF) and Group 2 (SDF + PI). (**a**,**A**) Preoperative images of a sectioned tooth showing carious lesions. (**b**,**B**) Preoperative micro-CT images showing radiolucent carious lesions. (**c**,**C**) Micro-CT images after application of SDF showing increase in the radiopacity of the carious lesions as well as silver precipitation on the external surface of the carious lesion (short white arrows). (**D**) Micro-CT images after application of PI showing increase in radiopacity likely due to the formation of silver iodide precipitation within the carious lesion (long white arrow). (**e**,**E**) Micro-CT images after 15 mL water rinse showing decrease in the radiopacity of the carious lesions compared to the same areas in (**c**,**D**) (outlined arrows). (**f**,**F**) One week post-operative images. (**f**) Dark staining of the carious lesion likely due to silver oxidation, and precipitation of silver at the junction of the affected and infected dentin (long black arrow), and penetration of the silver into the dentin tubules forming microwires (short black arrow). (**F**) Absence of dark stain in the Group 2 likely due to the formation of silver iodide.

**Figure 3:**
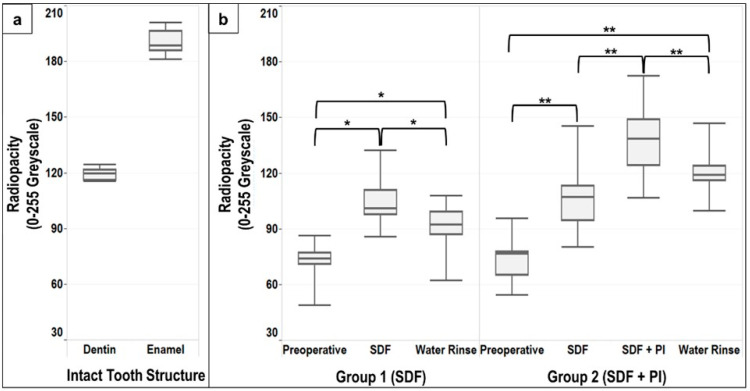
Descriptive analyses expressed as median, lower and upper quartiles, minimum and maximum greyscale values. Analyses are based on 0–255 greyscale. (**a**) Intact tooth structure includes dentin and enamel away from the carious lesions. (**b**) Group 1 includes descriptive analysis of the preoperative carious lesions, after SDF application and after rinse with 15 mL water. Group 2 includes descriptive analyses of the preoperative carious lesions, after SDF application, after PI application, and after rinse with 15 mL water. * *p* < 0.017 (adjusted *p* values for multiple comparisons in group 1), ** *p* < 0.008 (adjusted *p* values for multiple comparisons in group 2).

Pictures and descriptive analyses of the radiopacity of the intact tooth structure and the carious lesions expressed as median, lower and upper quartiles, minimum and maximum greyscale values are shown in Figure 2 and Figure 3.

A statistically significant difference (*p* < 0.005) was found between the radiopacity of intact dentin and the carious lesions in both groups, with no difference in the preoperative radiopacity of the carious lesions between Group 1 and 2 (Mann–Whitney U test *p* = 0.739) (Figure 3b).

Application of SDF alone resulted in a significant increase in the radiopacity of the carious lesions compared to the preoperative radiopacity in Group 1 and Group 2 (*p* < 0.017 and *p* < 0.008, respectively) (Figure 3b). After SDF application, no difference was found between the radiopacity of the carious lesions between Group 1 and Group 2 (Mann–Whitney U test *p* = 0.579). Overall, the radiopacity of the carious lesions increased by 30–45% following SDF application (Table 1). However, differences between intact dentin and the carious lesion after SDF application was still observed (Group 1 *p* < 0.05, Group 2 *p* = 0.114). Application of PI after SDF resulted in a significant increase in the radiopacity of the carious lesions in Group 2 (*p* < 0.008) (Figure 3b). This increase was approximately 80% compared to the preoperative radiopacity of the carious lesions (Table 1). Application of PI after SDF significantly increased (*p* < 0.05) the radiopacity of the carious lesion compared to intact dentin. However, enamel remained significantly (*p* < 0.005) more radiopaque than the carious lesions after SDF + PI application.

Although water rinsing after SDF and SDF + PI application resulted in a significant decrease in the radiopacity of the carious lesions in Group 1 and Group 2 (*p* < 0.017 and *p* < 0.008, respectively) (Figure 3b), a statistically significant difference was observed when comparing the preoperative radiopacity of the carious lesion to the radiopacity of the carious lesions at the end of the experiment in Group 1 and Group 2 (*p* < 0.017 and *p* < 0.008, respectively) (Figure 3b). Thus, the radiopacity of the carious lesions following water rinsing remained 29% higher compared to the preoperative lesions in Group 1, and 56% higher than the preoperative lesions in Group 2 (Table 1).

**Figure 4:**
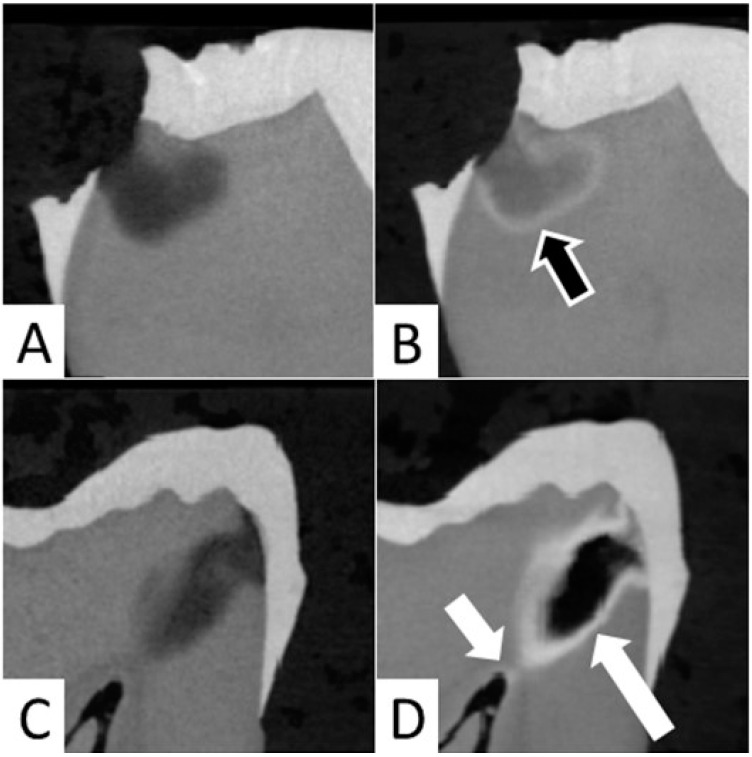
(**A**,**B**) Micro-CT images showing precipitation of SDF at the junction of affected and infected dentin (outlined arrow). (**A**) Preoperative image and (**B**) after SDF application. (**C**,**D**) Micro-CT image showing aggregation of SDF (white long arrow) and penetration of the SDF to the pulp chamber (short white arrow) (**C**) Preoperative image and (**D**) after SDF application.

In the current study, it was observed that application of SDF resulted in precipitation of silver at the junction of affected and infected dentin in 12 out of 20 samples (Figure 4A,B). In both groups, the formation of a large aggregation of SDF within the carious lesion was noted in four samples, and SDF reached the pulp chamber in cases of deep caries lesions in eight samples (Figure 4C,D).

Application of SDF alone resulted in dark staining on all teeth in Group 1. However, mild or no stain was noted in all carious lesions treated with SDF + PI in Group 2 (Figure 1).

## 4. Discussion

This report is the first to compare the change in the radiopacity of the carious lesions before and after application of SDF and PI. In addition, this is the first study to describe the impact of water rinsing on the radiopacity of the carious lesions treated with SDF and SDF + PI. Our investigation suggests that rinsing SDF and SDF + PI with water could reduce the SDF and SDF + PI content within the carious lesion. Although the manufacturer protocol indicates not to rinse with water after SDF application, the oral cavity is rich in saliva, which could be self-cleansing to the SDF and SDF + PI. As a result, multiple applications of SDF are likely needed to restore the SDF content within the carious lesion [5].

In clinical practice, many situations may require immediate restoration of the SDF-treated tooth with materials such as glass ionomer and/or composite resin restorations [10]. These materials frequently require application of a weak acid (e.g., 20% polyacrylic acid or 35–38% phosphoric acid), which should be rinsed away with water. Given that a washing step may decrease the content of SDF within the carious lesion but is needed to restore the bonding strength of restorative materials [6], there is a fine balance between the use of SDF and the longevity of bonded restorations.

Our findings suggest that SDF application increases the radiopacity of the entire carious lesions. This is likely due to the presence of silver ions [11]. However, other compounds such as Calcium Fluoride (CaF2) could also have formed and contributed to the change in radiopacity of the carious lesion after SDF application [7]. Furthermore, the radiopacity of carious lesions was increased significantly after PI application (Figure 2 and Figure 3b), likely due to the presence of radiopaque iodine and the formation of a white creamy silver iodine precipitation [8,12].

Occasionally, SDF seemed to reach the pulp chamber in deep carious lesions (Figure 4C,D). This is consistent with previous observations in primary teeth [13]. Dentin tubules are permeable channels that increase in density and diameter in close proximity to pulp [14,15]. Therefore, application of SDF to deep carious lesions could be associated with risk of SDF traveling through the dentin tubules (Figure 2f) and reaching the pulp chamber (Figure 4D), which could lead to pulpal inflammation and necrosis [16,17,18]. In our study, formation of dentin tubules micro wires could not be distinctly seen on micro-CT possibly due to the lower resolution (12 microns) compared to a prior study (1.3 micron) [19]. However, SDF penetration into dentinal tubules was seen on macro photographs of sectioned teeth (Figure 2f).

Our results are consistent with previous reports using SDF on primary teeth. Carious lesions are less radiopaque at the outside layer and increase gradually in radiopacity until sound dentin is reached [7]. This gradual transition poses a challenge in terms of successful separation between the affected and infected dentin using micro-CT images. Figure 2 shows also that affected dentin extends significantly beyond the distinct carious lesion on the micro-CT 3D reconstructions. This difficulty increased after SDF application as an overlap between the radiopacity of the carious lesion and intact dentin was noted after SDF application (Figure 3). Furthermore, previous reports suggested that the penetration depth of silver was larger than the depth of the distinct carious lesion [7]. This was based on the changes in the mineral density of the carious lesion before and after SDF application using a repositioning index for micro-CT scanning [7]. In our study, we attempt to reduce the overall variability by scanning the same samples and applying a coregistration technique to reposition the 3D reconstructed images and ensure that the region of interest is consistent throughout the experiment.

In the present experiment, application of SDF and SDF + PI occasionally resulted in large aggregates and very dense radiopaque layers (Figure 4C,D). This might be linked to the characteristics of the carious lesions, although no correlation was found between the presence of large aggregates and the preoperative radiopacity of the carious lesions (data not shown). Consequently, we opted to present the results as median and interquartile range to limit the impact of extreme values. In addition, although no significant difference was found when comparing the distribution of our data to normal distribution, we opted to report the results of non-parametric tests instead of the ANOVA repeated measures, as the Mauchly’s test of Sphericity tended to be statistically significant (Group 1 *p* = 0.077, Group 2 *p* = 0.089), which could violate the test assumptions. Regardless, similar results were obtained when using the ANOVA repeated measures and Wilcoxon Signed Rank Test with Bonferroni adjustment for multiple comparisons.

Finally, although the use of micro-CT as a nondestructive tool offered the advantage of analyzing the same sample before and after the application of SDF, SDF + PI and water rinsing, the results of this study should be interpreted with caution given the small samples size. In addition, the results are limited by the lack of elemental analyses, in vitro nature of the current experiment and the absence of intra-pulpal pressure. Future studies should include larger sample sizes and test the effect of applying PI individually on the carious lesions.

## 5. Conclusions

The radiopacity of the carious lesions increases after SDF and SDF + PI applications. Water rinsing seems to reduce the radiopacity of SDF- and PI-treated lesions.

## Figures and Tables

**Table 1:** Percentage increase of the preoperative radiopacity of the carious lesions after SDF application, PI application, and rinse with 15 mL water (values expressed as lower quartile (Q1), median and upper quartile (Q3)).

	After SDF Application			After PI Application			After Water Rinse		
	Q1	Median	Q3	Q1	Median	Q3	Q1	Median	Q3
Group 1 (SDF)	0.22	0.30	0.42				0.16	0.29	0.34
Group 2 (SDF + PI)	0.24	0.45	0.71	0.64	0.80	1.10	0.50	0.56	0.93

## Data Availability

Data is contained within the article. Further enquiries can be directed to the corresponding author.
